# Edge based distributed framework for real time hazard detection and road safety in smart transportation

**DOI:** 10.1038/s41598-026-42899-w

**Published:** 2026-04-13

**Authors:** Dinesh Sahu, Shiv Prakash, Vivek Kumar Pandey, Pratibha Dixit, Tiansheng Yang, Rajkumar Singh Rathore, Lu Wang, Yazeed Alkhrijah, Korhan Cengiz

**Affiliations:** 1 SCSET, Bennett University, Plot Nos 8, 11, TechZone 2, Greater Noida, Uttar Pradesh 201310 India; 2 Department of Electronics and Communication, University of Allahabad, Prayag Raj, Uttar Pradesh India; 3 King George Medical University, Lucknow, Uttar Pradesh India; 4 University of South Wales, Pontypridd, UK; 5 Cardiff School of Technologies, Cardiff Metropolitan University, Cardiff, UK; 6 Xi’an Jiaotong-Liverpool University, Suzhou, China; 7 Department of Electrical Engineering, Imam Mohammad Ibn Saud Islamic University (IMSIU), Riyadh, Saudi Arabia; 8 Department of Electrical-Electronics Engineering, Biruni University, Istanbul, Turkey; 9 Faculty of Information Technology and Systems University of Jordan, Amman, Jordan

**Keywords:** Edge computing, IoT, Real-time hazard detection, Smart transportation systems, Distributed framework, Machine learning algorithms, Vehicle-to-everything (V2X) communication, Computer science, Information technology

## Abstract

The main drawbacks of centralized smart-transportation pipelines are latency, bandwidth, and scalability limitations, which restrict the real-time detection and notification of hazards. Intended to develop and test a distributed edge structure capable of supporting low-latency, efficient hazard detection and disseminated alerts widely and quickly. We combine the edge-based IoT sensing (roadside units, in-vehicle devices, mobile crowdsensing) and local processing at the edge nodes. A combination of ensemble machine-learning (Random Forest, Gradient Boost) with Probabilistic Cellular Automata and Markov Decision Processes to predict hazards based on the speed, density, and environmental conditions of the traffic. An alert distribution layer (V2X, V2V, V2N) is one of the layers of the SUMO simulations, which benchmarks performance on centralized and scheduling benchmarks (RR, LC, FCFS, SJF, Random). The framework has a maximum hazard-identification precision of up to and including 95 percent when simulated, can reduce its alert latency to 0.2 to 0.3 seconds (compared to a baselines minimum of 0.8 to 1.5 seconds), consumes less energy, and balances its loads (lowest 0.04-0.7-sigma edge-node load) and achieves high throughput (27 to 30 tasks/s) with exceptionally good scalability and low drop rates. A V2X distributed edge architecture with V2X and hybrid ML/PCA/MDP analytics can provide accurate, low-latency hazard detection and alerts, and is superior to centralized methods, offering a practical foundation for safer and more resilient transportation in both urban and rural settings.

## Introduction

The overall increase in the rate of urbanization and number of vehicles has prompted traffic difficulties towards the achievement of safety on roadways^1,2^. Challenges such as hour-long junction congestions and inadequate traffic data to support complex traffic analysis have been solved by smart transportation systems through IoT and advanced computing technologies. Such systems are meant to improve transportation operations, resolve congestion problems and prevent or minimize road accidents through analysis of data and making rational and intelligent decisions. However, existing centralized systems suffer from high latency, restricted bandwidth and scalability challenges, which makes them unsuitable for actual time hazard identification and detection^3,4^. The frequent use of IoT devices in smart transportation opens new alarming scenarios such as: vulnerability attacks whereby data integrity is threatened as well as the integrity of the devices hitherto applied in the detection of hazards are themselves attacked and disabled. Also, the scalability of the system through the application of the virtualization techniques has been improved while, on the other hand, the IoT security issues that are newly arising must be well protected.

### Motivation

Innovations in smart transportation systems have observed a huge demand in the recent past because of more incidence of road accidents and the rising need for traffic control. Current research shows that traffic congestion amounts to several billions of dollars and is a main source of pollution of the physical environment^5^. Centralized architectures that are common in conventional implementations depend extensively on cloud infrastructures for data processing and analysis, which degrades the system’s capability for real-time operations, including hazard identification and warnings. They also have issues with information congestion when dealing with data handling produced by IoT apparatus in transportation systems. The majority of the current smart transportation systems are dependent on the cloud-centric architectures, which are characterized by a high latency, bandwidth, and lower scalability. Contrary to that, the suggested distributed model exploits edge-based IoT incorporation with V2X communication to provide the localization of decision-making, thus lowering the detection latency and guaranteeing high scalability in heterogeneous networks.

On the other hand, distributed edge computing frameworks are a more viable solution as these frameworks address data processing in real-time, near the source, and can be easily scaled^6^. Edge nodes can perform data pre-processing, hazard forecasting, and disseminating alerts within microseconds making them suitable where and when transport environments are volatile and diverse. Although numerous studies have been conducted on cloud-based and edge-assisted smart transportation schemes, the majority of the current methods mainly focus on traffic optimization, congestion control, or massive data aggregation and still heavily depend on the centralized or semi-centralized decision-making architectures. They are commonly not well-matched to any safety-critical system that requires ultra-low latencies, local intelligence, and also real-time response, especially in real-time hazard identification and alert broadcasting. Moreover, existing research literature generally considers each of the three domains individually, i.e., machine learning-based traffic analysis, spatio-temporal traffic modeling, or the scheduling and communication strategies. The collective control of the dynamic process of spatio-temporal traffic, the use of uncertainty in making decisions, and the distribution of alerts through V2X in a single, fully distributed system-level structure is virtually unexplored. To address further such gaps, this paper introduces a distributed edge-based hazard detection architecture that integrates ensemble machine learning, Probabilistic Cellular Automata, and Markov Decision Processes with V2X communication, to provide suitable, low-latency, and scalable hazard detection and alerting to the next-generation smart transportation system.

### Problem statement

Therefore, there is a significant demand for a distributed, low-latency hazard detection mechanism that can overcome the problems stemmed from centralized approaches. In extensive approaches do not satisfy the tight real-time constraints implanted in today’s transportation systems. The outlined challenges imply the need for the IoT, edge computational platforms, and powerful algorithms to implement intelligent systems to achieve safe traffic flow^7^.

In order to increase the clarity, we now clearly state the problem we intend to solve in the given work in the form of developing a distributed hazard detection and alert-dissemination framework that can work in reality with under latency constraints, where the traffic conditions are not homogeneous, and the conditions of communication are of uncertain reliability. It aims to simulate traffic behavior, identify threats in a precise way, and optimize the delivery of the alerts by reducing the time of detection, expanding the space area, and maintaining the system stable in different density conditions, noise, and movement patterns. This sophisticated model makes the computational objectives, constraints and performance indicators behind the proposed solution clear.

### Contributions

1. *Distributed edge-centric hazard detection framework:* This paper presents a full distributed and edge-based architecture of smart transportation systems to detect and mitigate hazards in real time. The framework closely combines the IoT sensing, localized edge intelligence, and V2X communication to facilitate low-latency, scalable, and safety-oriented decision-making without the need to call upon the centralized cloud computing.
2. *Hybrid analytical and decision-making model for traffic risk assessment:* It presents a hybrid modeling framework based on the synergy of ensemble machine learning with Probabilistic Cellular Automata and Markov Decision Processes to jointly learn traffic dynamics at the spatio-temporal scale and the uncertainty-aware decision-making. Such integration makes the correct identification of hazards and adaptive dissemination of alerts possible during heterogeneous and dynamic conditions of traffic flow.
3. *Comprehensive system-level evaluation and benchmarking:* The suggested framework is thoroughly tested with the help of large traffic simulations and actual mobility traces, taking into account the main performance indicators, including the quality of hazard detection, the delay of receiving the alerts, the use of resources, energy consumption, and load balancing. The comparison of the results with centralized and baseline methods of scheduling proves to be consistent with the increase of responsiveness, scalability, and efficiency of the system.

### Structure of the paper

The remainder of this paper is organized as follows: “Related work” section provides the literature study of some previous works that use IoT for hazard detection and edge computing in the transportation system. “Proposed framework” section outlines the architecture of the designed distributed edge framework as well as its constituents and workflow in detail. “Mathematical modeling for real-time hazard detection framework” section illustrates the Mathematical Modeling for Real-Time Hazard Detection Framework. “Experimental setup” section discuss the experimental setup used in this research. “Results and discussion” section contains the experimental results and comparison analysis of the proposed system. “Pilot testing as future work” section 7 presents the conclusion and a discussion on the future research opportunities.

## Related work

Smart transportation systems have been an area of interest in research in traffic management and road safety employing cloud-computing, IoT, and edge-computing algorithms. Some current issues include substantial progress in the area but lack of robust methods for the intuitive identification of real-time hazards and alarms. Hazard detection centralization systems majorly involve cloud-based structures where data regarding traffic is collected, processed, and analyzed. These systems improve by their capacity in coordinating and operating big data sets on geophysical areas. But they fail in real-time applications by having serious drawbacks as follows. The characteristic delay in transmitting data from sensors, to cloud server and back to the consumer circuit is a major challenge in IoT especially in time critical tasks such as sending of hazard alerts. In addition, cloud systems have throughput limitations during peak usage or network outages, which limits cloud reliability^8,9^. Systems that are centralized are able to quickly identify excess traffic because their connections are primarily based on the high bandwidth networks^10–12^. In the same way, Cloud architectures can generally handle large-scale data well, but when there are more frequent and update requests, their response time can be relatively slow^13–15^. For that reason, these challenges call for even more distributed and localized solutions. In transportation systems, the implementation of IoT solutions has brought changes through acquiring updates on the traffic situations, how vehicles function, and other limiting aspects of whatsoever area. Intelligence of Things sensors in automobiles as well as transport systems gather and relay information for traffic control, predictive repairs, and event monitoring^4,16,17^.

An IoT-based framework of task scheduling is designed in edge to analyze its applicability to relieve congestion and enhance flow^18–20^. The application of IoT in machine-learning-using route finding emphasizing its reduction of accident and increase of safety^21^. However, IoT systems are usually insufficient in terms of computational power to process information and perform hazard detection locally, thus, their applications typically involve interaction with edge or cloud computing^22^. Edge computing becomes an ideal solution for solving the problems of centralized systems and enhancing the application of IoT in smart transportation. Edge computing helps to process data closer to where it is generated thus eliminating latency while at the same time improving the efficiency of real-time applications^23,24^.

For instance, the evidence that edge-based systems are beneficial for traffic management to enhance response times for hazard alerts^3,25^. Typically, these frameworks employ machine learning algorithms to analyse data from the sensors thereby making decisions locally at a fast pace. For example, the RACE framework proposed to connects edge nodes in IoT devices which makes it possible for scheduling and load balancing in cloud-fog architecture to be inexpensive^26–28^. Nevertheless, the majority of the ECSs are designed to optimize general traffic, with little consideration given to real-time hazard identification. Discuss edge-based frameworks in smart transportation. Even though centralized and IoT-based solutions have solved a number of problems related to traffic management, the aspects of scalability and real-time response are still apparent^29,30^. A centralized approach cannot come closer to the real-time detection of hazards while on the other hand, IoT based systems may need to depend on other computation resources^31,32^.

The reviewed frameworks show the potential of enhancing edge computing real-time performance; however, no framework is exclusively designed for distributed hazard detection systems^6,33,34^. Past works lack insights into the blending of superior algorithms like Probabilistic Cellular Automata and Markov Decision Processes in the processes of risk identification^35–38^. Also, interaction models such as Vehicle-to-Everything (V2X) concerning the delivery of a hazard alert is still an unexplored area^39–42^. These gaps suggest the need to develop distributed edge frameworks tailor-made for improving real-time detection and alarm systems for hazards.

In order to further emphasize the novelty of our method, we have broadened the discussion of the comparison of our framework with the edge-based and cloud-centric hazard detection architectures. The existing systems are typically based on centralized processing by the clouds, single-model based learning pipelines or heuristic-based decision-making rules that restrict their flexibility and real-time performance in changing traffic scenarios. In contrast to these solutions, our solution combines ensemble machine learning to hazard detection, Probabilistic Cellular Automata (PCA) to model traffic-flow evolution and Markov Decision Processes (MDP) to optimally plan hazard-responses in a single distributed edge architecture^11,43–45^. This combination is not discussed in the previous literature and allows simultaneously enhancing detection accuracy, alert diffusion latency, and scalability of the system without being weak in various urban and semi-urban traffic conditions.

## Proposed framework

The conceptual model for real-time sensing of hazards in Smart transport includes applicability of IoT devices, edge computing and enhanced communication protocols for efficient mining of traffic data. Unlike the centralized system, this distributed system carries out computation at the edge nodes, hence, eliminate latency and bandwidth consumption while at the same time scale. Vehicle ECUs, roadside sensors, and smart phones serve as data providers that effectively collect data about traffic conditions, vehicle dynamic variables and environment state. This data is later analyzed at edge nodes through efficient and complex algorithms like Probabilistic Cellular Automata (PCA) & Markov Decision Processes (MDP) for effective and real-time hazard identification. The framework further also uses Vehicle-to-Everything (V2X) for real-time conveying of hazardous alert information to vehicles, traffic operators, and emergency response systems. This multifaceted design guarantees an effective and fault-tolerant system to overcome the flaws of a centralized system when seeking improvements in road safety and traffic organization.

Unlike traditional cloud-based systems that rely on the remote server to do their computations and make decisions, the given framework uses distributed processing on the edges, along with V2X communication, to reduce latency and bandwidth usage. The system facilitates computation at the edges, meaning that the physical infrastructure is no longer needed within a centralized location as the edge nodes process computation, hence removing cloud bottlenecks and alleviating the risks of sluggish hazard detection when overloaded. V2V, V2I, and V2N protocol integration guarantees the rapid and reliable dissemination of alerts to the heterogeneous environment, which makes the framework more scalable, resilient, and better-suited to real-time smart transportation applications.

The choice of algorithms in the suggested framework was informed by the necessity to strike the balance between accuracy, flexibility and scalability in the context of smart transportation. Random Forest and Gradient Boost were used as ensemble learning techniques as they are very effective when dealing with the heterogeneous and noisy traffic data and they outperform the single classifiers like decision trees or logistic regression. Probabilistic Cellular Automata were to be included that exhibit the spatio-temporal characteristics of the traffic flow and local vehicle interactions that are hard to model with traditional regression or queuing models. The Markov Decision Processes were incorporated to deal with sequential decisions under uncertainty especially in hazard detection and alert dissemination where the heuristic or rule-based methods are not always able to respond to the changing circumstances. The framework is more complementary by integrating accurate hazard detection of the ensemble models, realistic evolution of the traffic by cellular automata, and optimal alert dissemination through decision processes, which makes it stronger, scalable, and efficient than the traditional single-model solutions.

### System architecture

**Fig. 1:**
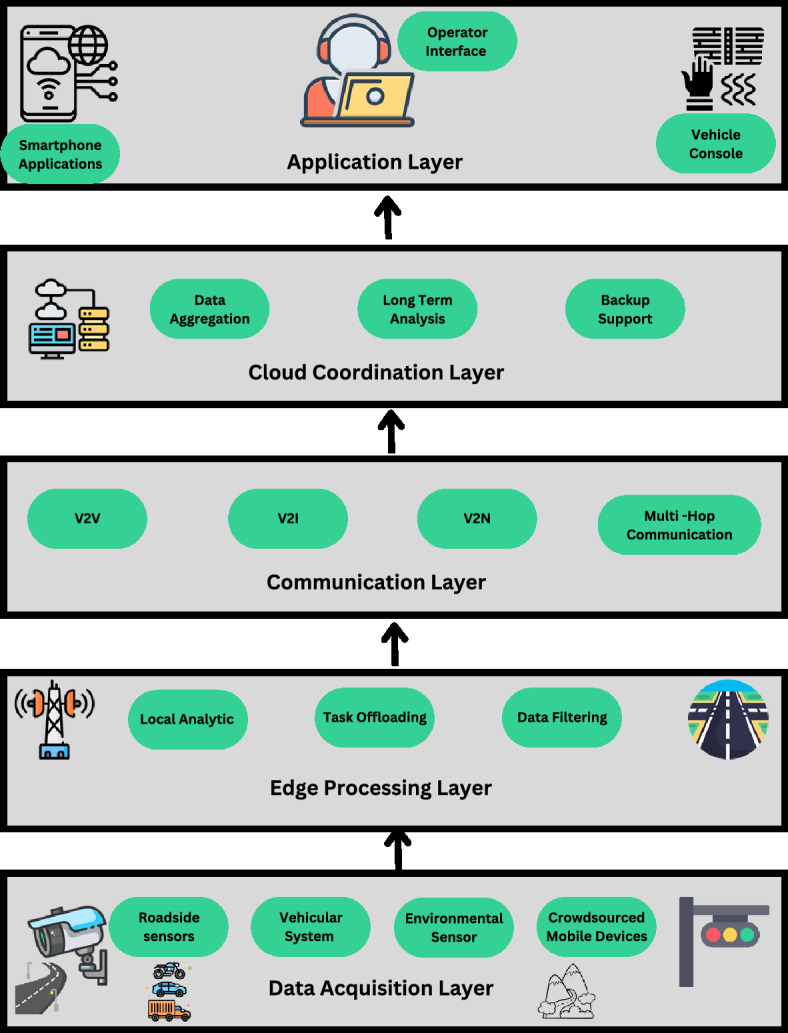
Overall architecture of the proposed edge-based hazard detection framework.

This setup of the real-time hazard detecting framework for smart transportation systems is the Multiple Layer System and each of the layers plays important roles to give low-latency, scalable, efficient hazard management as shown in Fig. 1.

The layers are as follows:

1. *Data Acquisition Layer:* The IoT Data Acquisition Layer is an initial layer of the framework because it provides real-time information from different transport network sensors and devices. They include roadside sensors, vehicular systems, environmental sensors, and portable machines. This layer identifies relevant features including velocity, distance, climate and traffic congestion levels. Thus, it also allows collecting data from the crowds through their mobile devices and improving the system’s reliability and coverage. For instance, cameras are able to sometimes identify obstructed roads or an accident that has occurred, and accelerometers in vehicles may be able to detect cases of sudden braking. This layer involves acquiring of integrated information that is vital for the proper identification of hazards and the prognosis of their occurrence.
2. *Edge Processing Layer:* IoT data is processed locally at the Edge Computing Layer at different edge nodes – for instance, intersections, cellular towers or RSUs. This layer minimizes the dependency on centralized cloud computing to enable efficient and low latency analytics by leveraging on spare bandwidth space. It utilizes sophisticated and intelligent analyses of traffic data through PCA and MDP so as to identify risks en route in realtime. The use of mechanisms for offloading tasks provides for optimal use of available computing resources; decentralization of the decision-making process allows reacting to threats instantly. For example, edge servers may be used to filter through video streams to look for signs of collisions or to analyze data residing at different locations in order to determine the most effective traffic patterns.To prevent oneself from vulnerability attacks the edge processing layer involves encrypted layers together with secure virtualization layers. All these measures help guarantee the local traffic analytics as well as task offloading processes, which in turn improve the IoT security within distributed settings.
3. *Communication layer:* The Communication Layer makes information exchange within the framework fast and free from high latency using Vehicle-to-Everything (V2X). These protocols include Vehicle-to-Vehicle (V2V), Vehicle to Infrastructures (V2I), and Vehicle to Network (V2N). This layer communicates real time hazard information to other vehicular units, traffic management systems, and first responders. Multi-hop communication means that alerts can be sent over a greater distance, so even if a distant car is involved, it will receive the update. For instance, this layer allows the informing of vehicles about any abrupt hindrances on the road or the coordination of vehicles regarding traffic congestion in hazard areas.
4. *Cloud Coordination Layer:* The Cloud Coordination Layer carries out the high-level data collection and storage from around the world and performs long-term analysis. It accumulates and organizes data from many edge nodes to produce an overall picture of traffic and risk on large areas. This layer is especially useful for practitioners involved in the construction of predictive models, infrastructure requirements, and/or trend analysis spanning numerous years. Also, it acts as a recovery tool in the case of specific edge node failures to maintain the system’s stability. Such uses include creating traffic heat maps for countries to avoid traffic congestion and analysis of the past to locate areas with high risk in order to develop more infrastructure.
5. *Application layer:* The Application Layer also known as the interface layer is the part of the framework that interacts with the user and presents him with user friendly tools to access information on current hazards and/or helps him navigate the environment. It comprises of smart phone applications, vehicle consoles and operators’ Interfaces whereby users can easily interface the system. The drivers receive information particularly regarding the hazards encountered on the road receive information on, safe driving routes and report more cases that are incorporated into the feedback loop of the system. Incidents can be centrally managed and responses coordinated well by the advanced traffic operating dashboards. For example, some of the areas may include safe evacuation routes in emergencies handled by the apps of mobile devices and efficient incident management by operator interfaces.

### Workflow

**Fig. 2:**
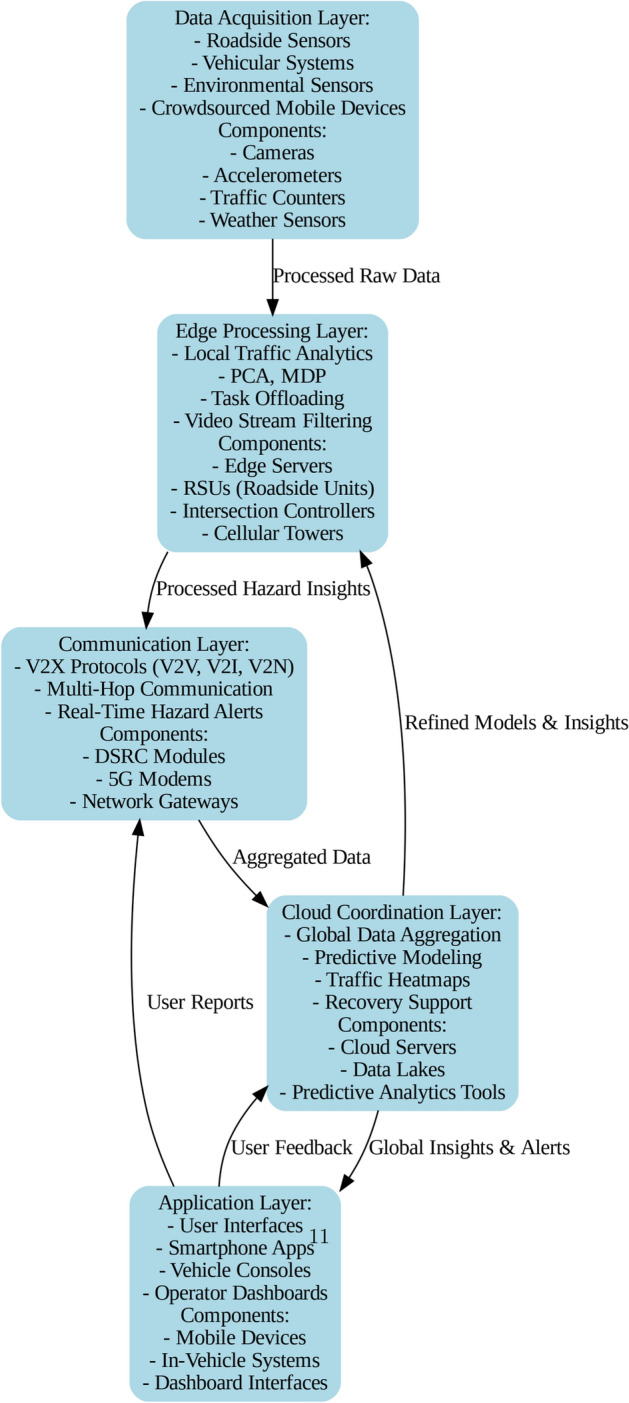
Workflow of the hazard detection and alert dissemination pipeline.

Figure 2 shows a hierarchical integrated system model for identifying hazards and responding in smart transportation systems. Starting at the Data Acquisition Layer where raw data is gathered from the infrastructure level like roadside units, vehicles, environment sensors, and from the civil level like Mobile Applications of smart phones and social media shares. Various parts that are involved in anything from the inside and outside cameras, accelerometers, traffic counters, weather sensors and much more, ensure all round coverage and collection of information about traffic and its surrounding environment. This raw data is then preprocessed and sent to the Edge Processing Layer for Probabilistic Cellular Automata (PCA), Markov Decision Processes (MDP), and other analytics, and task offloading mechanisms. Remarakable components including edge servers, RSUs, intersection controllers, Ad-hoc base stations, and cellular towers facilitate the processing and filtering of the hazards in video streams. The processed hazard insights are then conveyed to the Communication Layer which employ V2X protocols which include Vehicle-to-Vehicle, Vehicle-to-Infrastructure, and Vehicle-to-Network with multi-hop messages to broadcast real-time hazard alert. The low-latency, high-reliability communication is made possible with devices such as the DSRC modules, 5G modem and network gateways. Employer-level data and users’ reports go to the Cloud Coordination Layer where worldwide data accumulations, prognostic account, and traffic heat maps are produced. This layer enables recovery activities involving substrates such as cloud servers and data lakes alongside using big data analytics weapons for enhanced decision making. Last but not least, the Application Layer provides connections between end-users via a smartphone app or vehicle console and operator’s central console. Aspects such as a mobile terminals, car’s built-in systems, and dashboard displays offer global information and notifications and gather user data for further improvement of the system. This interaction enables effective identification of hazards, immediacy in relaying alerts and consequent improvements on the system, which significantly address issues of road safety and traffic regulation.

To enhance the clarity of methods, as well as guarantee the possibility to reproduce the results, this section will thoroughly describe the entire hazard detection pipeline and the mechanism of the spread of warnings. The framework starts with the gathering of data with the support of heterogeneous IoT sources, such as roadside sensing units, in-vehicle equipment, and mobile crowd-sourced inputs. At the edge, every data stream is pre-processed to eliminate noise, synchronize the timestamps and detect the most important features of a vehicle, including vehicle speed, acceleration trends, vehicle density, position variability, and the environmental conditions. These are the features obtained that are used as input to the ensemble learning module which combines the use of Random Forest and Gradient Boosting classifiers. The ensemble enhances the strength by making a decision across various weak learners, which facilitates the backbone hazard detection in a noisy and dynamic traffic environment.

After the preliminary categorization, a Probabilistic Cellular Automata (PCA) model is used to model the spatio-temporal traffic behavior. The PCA represents each road segment as a cell and transition rules change the states of cells depending on the interactions between vehicles, the changes in the flow of traffic, and detected anomalies. This enables the system to learn the hazard trends that have not been anticipated like wave congestion or reduced braking or acceleration. The result of the PCA is inputted into a Markov Decision Process (MDP) that is a model that describes the decision making under uncertainty. The MDP takes into consideration definitions of states (traffic states), actions (alert decisions), transition probabilities, and reward structure in order to identify optimal actions to mitigate the hazard and share alerts. The computed decision made by the MDP at that point activates a multi-channel alert distribution layer which makes use of V2V, V2I, and V2N communications. The importance of this layered design is that alerts are sent with limited latency even when in environments where connectivity varies or have many vehicles.

Ensemble learning models are used due to the fact that they offer greater robustness and generalization to heterogeneous traffic inputs than single models. The inclusion of Probabilistic Cellular Automata is motivated by the fact that local, decentralised and stochastic interactions inherent in real world traffic flow are naturally captured using Probabilistic Cellular Automata. Markov Decision Processes are not omitted since they can be used to make a sequence of decisions under uncertainty that is needed to make the best decisions about hazards-response planning. A combination of these elements creates a single architecture, which mitigates the weaknesses of cloud-centric and heuristic methods by offering scalability, flexibility, and low-latency responsiveness.

### Methodological rationale

The methodological design of the proposed framework will be motivated by the necessity to assist in the accurate, low-latency, and adaptive hazard detection of the transportation environment that is highly dynamic and safety-critical. The choice of techniques is inspired by their complementary advantages, as well as their appropriateness to the heterogeneous, uncertain, and spatio-temporal data on traffic.

Ensemble learning techniques namely Random Forest and Gradient Boosting are used in hazard detection because they are effective in dealing with noisy, high-dimensional and heterogeneous data produced by IoT sensors and vehicular networks. As opposed to single classifiers, ensemble models can reduce variation and enhance generalization by combining multiple learners, and would hence be effective in real world traffic settings where sensor errors, dropouts as well as sudden changes of behavior are routine. They are also more effective in detecting non-linear feature interactions, which increases their capacity to detect under different traffic and environmental conditions.

To simulate the spatio-temporal dynamics of traffic, Probabilistic Cellular Automata are embraced since they are inherently localized interaction of vehicles and road segments. The dynamics of traffic flow are both spatial and time-dependent and are dependent on the behavior of the neighboring vehicles, topology of the road, and the environment. PCA helps the framework to serve these localized dependencies and stochastic transitions more effectively than conventional queuing, regression, or entirely data-based models and thereby offers realistic traffic state development to conduct hazard analysis.

The Markov Decision Processes are integrated to facilitate sequential decision processes in the face of uncertainty especially in hazard response and alert propagation. In dynamics transportation, alerting decisions require taking into account the current state of the system as well as the consequences which are going to happen in the future, including the delay in communication, load on node, and proximity of the vehicles. MDPs present a principled model to approximate the following trade-offs and to create policies that maximize the timing and coverage of alerts and minimize the latency and consumption of resources.

All of the approaches mitigate a particular shortcoming of the methods that are used individually: ensemble learning provides proper hazard detection, PCA makes use of traffic dynamics, and the MDP supports the optimal decision-making processes. Their combination into a coherent and scalable framework based on edges give solutions to real-time hazard detection and alert broadcasting in intelligent transportation systems.

## Mathematical modeling for real-time hazard detection framework

The proposed framework can be developed in a mathematical model dealing with IoT data acquisition, edge processing and dissemination of hazard alert. This mathematical model is directly associated with the operational aspects of the suggested distributed edge structure. All the variables and objective functions are implemented in the edge processing, communication, and application layers as explained below. The following is the mathematical model along with description of each component of the model and the pertinent equations. The system has following components

Let $\textbf{D} = \{d_1, d_2, \ldots , d_n\}$ represent data streams from $n$ IoT sensors where $d_i$ is the data from sensor $i$ (e.g., traffic flow, speed, environmental conditions).Let $\mathcal {E} = \{e_1, e_2, \ldots , e_m\}$ represent $m$ edge nodes that process data locally.Let $\mathcal {V} = \{v_1, v_2, \ldots , v_k\}$ represent $k$ vehicles or users in the system. Each vehicle $v_j$ receives alerts based on its proximity to hazards. Communication between components is modeled using$\mathcal {C}_{ij}$: Communication link between edge node $e_i$ and sensor $d_j$ and $\mathcal {C}_{i}^\text {V2X}$: Communication link between edge node $e_i$ and vehicles in its range. Let $\textbf{S}(t)$ represent the real-time state vector of the transportation system at time $t$, derived from sensor data. $\textbf{H}(t)$ represents the hazard state vector, where $h_i(t) \in \{0, 1\}$ indicates whether a hazard is detected at sensor $i$ (1: Hazard detected, 0: No hazard). $\textbf{A}(t)$ is the alert vector, where $a_j(t) \in \{0, 1\}$ indicates whether an alert is sent to vehicle $v_j$ at time $t$.

Each edge node $e_i$ processes data $\textbf{D}_i$ from its connected sensors and computes $h_i(t)$:

1 $$\begin{aligned} h_i(t) = {\left\{ \begin{array}{ll} 1, & \text {if } \mathcal {F}_i(\textbf{D}_i(t)) \ge \theta , \\ 0, & \text {otherwise}, \end{array}\right. } \end{aligned}$$

where: $\mathcal {F}_i(\textbf{D}_i(t))$ is the hazard detection function (e.g., anomaly detection, threshold comparison). $\theta$ represents hazard threshold value. Computation based on the threshold is used in a case where the data $d_j(t)$ is a numerical value, for instance, speed, temperature or traffic density. The function $F_i(d_j(t))$ is determined by comparing the data against a predefined threshold, $\theta$, and is computed as follows:

2 $$\begin{aligned} F_i(d_j(t)) = {\left\{ \begin{array}{ll} 1, & \text {if } d_j(t) \ge \theta , \\ 0, & \text {otherwise}. \end{array}\right. } \end{aligned}$$

Here, $\theta$ stands for the predetermined hazardous value measured to identify a form of danger for instance high speed value. It allows you to easily and quickly identify conditions that are at or above the crucial level that characterizes a critical state.

For each vehicle $v_j$, the edge node evaluates:

3 $$\begin{aligned} \text {distance}_{ij} = \sqrt{(x_j - x_i)^2 + (y_j - y_i)^2} \end{aligned}$$

where $(x_j, y_j)$: Location of vehicle $v_j$ and $(x_i, y_i)$: Location of hazard $h_i(t)$.

An alert $a_j(t)$ is sent to vehicle $v_j$ if:

4 $$\begin{aligned} a_j(t) = {\left\{ \begin{array}{ll} 1, & \text {if } h_i(t) = 1 \text { and } \text {distance}_{ij} \le R \\ 0, & \text {otherwise}, \end{array}\right. } \end{aligned}$$

where $R$: Communication range of edge node $e_i$.

The load $L_i(t)$ of an edge node $e_i$ is given by:

5 $$\begin{aligned} L_i(t) = \sum _{j \in \mathcal {V}_i} f_j(t) \end{aligned}$$

where $\mathcal {V}_i$ is vehicles handled by edge node $e_i$ and $f_j(t)$ represents computational demand for processing data for vehicle $v_j$. The objective is to minimize the load imbalance $\sigma _L$ across all edge nodes:

6 $$\begin{aligned} \sigma _L = \sqrt{\frac{1}{m} \sum _{i=1}^m (L_i(t) - \bar{L}(t))^2} \end{aligned}$$

where $\bar{L}(t)$ is the average load. The total latency $T_\text {alert}$ for hazard alerts is minimized:

7 $$\begin{aligned} T_\text {alert} = T_\text {process} + T_\text {comm} \end{aligned}$$

where $T_\text {process}$ is processing time at edge nodes and $T_\text {comm}$ is communication delay to vehicles.

Table 1 summarizes the way the proposed mathematical formulation can be directly mapped into the realized elements of the distributed edge-based framework.

**Table 1:** Mapping between mathematical formulation and implemented system components.

Mathematical element	Description	Implemented in
*H*(*t*)	Hazard state vector indicating the presence or absence of hazards at time *t*	Edge Processing Layer (Hazard Detection Module)
*A*(*t*)	Alert decision vector specifying whether alerts are issued to vehicles at time *t*	V2X Communication Layer (V2V, V2I, V2N)
$T_{\text {alert}}$	End-to-end hazard alert latency, including processing and communication delay	SUMO Simulation with TraCI Interface
$\sigma _L$	Load imbalance across edge nodes reflecting resource distribution efficiency	Edge Node Scheduler and Task Offloading Module
Coverage	Ratio of vehicles successfully alerted within the hazard zone	Application Layer (Vehicle Consoles and Mobile Applications)
$L_i(t)$	Computational load at edge node *i* at time *t*	Edge Processing Layer (Resource Management Unit)
$distance_{ij}$	Distance between hazard location and vehicle *j*	Edge Processing and Communication Layers
$\theta$	Hazard detection threshold dynamically updated using feedback	Edge Analytics and Model Update Module

The system dynamically updates its hazard state $H(t)$ and adjusts alerts $A(t)$ based on two key inputs: Driver feedback and sensor updates. Driver feedback, described by the function $F_j(t) \in \{0, 1\}$ reflects whether a driver saw a hazard or not at time t with 1 meaning he did. Thus, real-time sensor update, $D(t)$ makes constant data available for enhancing the system’s knowledge of hazardous conditions and notifications.

The goal of the proposed system is to perform hazard detection as accurately as possible with limited false positives. This includes the correction of the methods developed for maximum effectiveness in the identification of true hazards with the minimum possible errors. Another major goal is to reduce the amount of data exchange in the network between the edge nodes and central servers as a measure of optimizing the system’s efficiency. Further, the system proposes to enhance safety of vehicles whereby the noted strategies will focus on alerting vehicles with greater probabilities to collision particularly through their assessment of risk probability as influenced by their distance with the perceived threat. These objectives when achieved in their entirety provide the basis for according a sound, dependable, and safe hazard identification and warning in smart transportation networks.

1. Maximize Hazard Detection Accuracy
  8 $$\begin{aligned} \max \text {Accuracy} = \frac{\text {True Positives}}{\text {True Positives} + \text {False Positives}} \end{aligned}$$
2. Minimize Communication Overhead
  9 $$\begin{aligned} \min \text {Overhead} = \sum _{i=1}^m \mathcal {C}_{i}^\text {V2X}. \end{aligned}$$
3. Optimize Vehicle Safety
  10 $$\begin{aligned} \max \text {Safety Score} = \sum _{j=1}^k a_j(t) \cdot \text {distance}_{ij}^{-1} \end{aligned}$$
4. Minimize Hazard Alert Latency
  11 $$\begin{aligned} T_{\text {alert}} = T_{\text {process}} + T_{\text {comm}} \end{aligned}$$
5. Minimize Load Imbalance Across Edge Nodes
  12 $$\begin{aligned} \sigma _L = \sqrt{\frac{1}{m} \sum _{i=1}^m (L_i(t) - \bar{L}(t))^2} \end{aligned}$$
6. Maximize Alert Coverage
  13 $$\begin{aligned} \text {Coverage} = \frac{\text {Total Vehicles Alerted}}{\text {Total Vehicles in Hazard Zone}} \end{aligned}$$

The feedback loop entails obtaining feedback $F_j(t)$ about the credibility of the alerts from the vehicles. The above feedback is then used to adjust the parameter $\theta$ and improve further the hazard detection models that are used in the system and hence improving the accuracy of the alerts generated.The process includes previous steps to ensure for every time step $t$, until there are no more data to analyze or all risks are managed prohibitive.

This mathematical model offers a paradigm for both the deployment and the assessment of the how well the entire framework works in different research scenarios.

### Algorithm:Hazard detection and alert system

This algorithm 1defines how the alert of a hazard occurring in the smart transportation network can be identified in real-time and informed to the relevant parties. It starts by aggregating multi-source data from the IoT devices which includes the road sensors, vehicles, and environment systems. Information is preprocessed at the edge nodes, on the basis of PCA and MDP to identify the probable dangers. Identified risks are shared with other vehicles and the infrastructure through the communication layer using V2X interface for fast forwarding of the information to the immediate environment. They live in the cloud where the system adjusts its predictive models and traffic information depending on feedback it receives from end users regarding the level of detection accuracy and response time.

**Algorithm 1:**
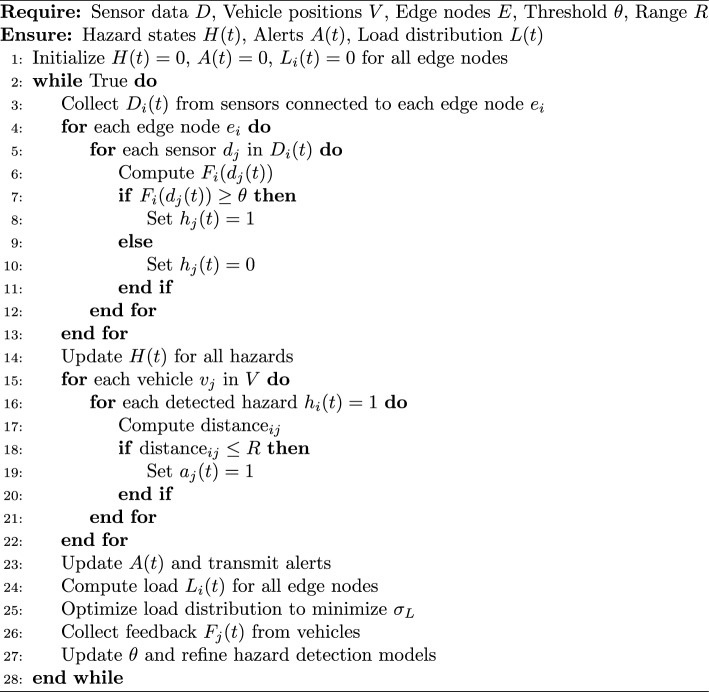
Hazard detection and alert system

To have a realistic and reproducible assessment of the proposed hazard detection and alert dissemination structure, this study uses well-specified parameter settings and system level assumptions applicable in cloud-fog based smart transportation settings. The traffic in the SUMO simulator is simulated over the following density ranges; low (50 −100 vehicles), medium (150 −250 vehicles), and high congestion (300 −450 vehicles), with the vehicle speed ranging between 20 −60 km/h under different road classes and peak/off peak conditions. Environmental conditions take into consideration some normal daylight situations where changes in visibility are implicitly represented through sensor noise parameters of roadside and in-vehicle units.

The assumptions made on communication are multi-channel V2V, V2I, and V2N communication based on latency models based on empirical research, where end-to-end delays are 5120 ms in the case of V2V, 1040 ms in the case of V2I, and 50120 ms in the case of V2N links. To simulate realistic wireless effects of interference and congestion, a drop probability of 1–3% is introduced in a packet drop. The base edge infrastructure of the system consists of 2.4 GHz quad core-based, 4 GB RAM, low energy budgeted fog nodes to emulate the practical deployment limits of roadside nodes. The cloud back-end servers are considered to have extensive computing power with insignificant processing delay when compared to the communication delays. The combination of these assumptions is an experimental setup that can be described as a controlled but realistic scenario to test algorithmic performance in terms of accuracy of hazard detection, response time, reliability, and scalability.

## Experimental setup

The experiments are performed using SUMO–Simulation of Urban Mobility: an open-source traffic simulation environment to study smart transportation systems. The test involved a wide range of conditions, such as intersections in urban areas, highways in rural and semi-urban areas, and the test was conducted in peak and off-peak conditions. The communication parameters were diverse to cover both short-range V2V communications and long-range V2I and V2N communications. To be validated, the SUMO simulation experiments were done with a set of about 15,000 vehicle traces simulated on 35 km of the road networks of urban, semi-urban, and highway topologies. The simulations were 60 minutes long with low (50 vehicles/km) to high congestion (250 vehicles/km) traffic densities. Besides that, real GPS data was used, belonging to an open dataset of 500 vehicles covered during 120 hours of driving, and the sampling was 1-second long. This combination of large scale synthetic traces and real world mobility measurements allowed the evaluation to account not only for experimental diversity but also for real-world vehicular dynamics. To obtain the strength of results, simulation scenarios were replicated 10 times with varying random seeds in order to consider stochastic changes in vehicle arrival, mobility models, and communication incidences. The reported performance measures constitute the averages of the values in these runs, and standard deviations are also calculated to reveal variability and consistency. To provide a complex check of the hazard detection algorithms, the simulation environment features a wide variety of instances. Urban dynamics comprise traffic congestion at rush and other times, intersections with traffic signals, roundabouts, and diverse road categories so that effects of congestion can be judged. Rains, fog, snow, and fogs are mimicked under the varying parameters including rain rate, wind speed, and visibility to evaluate the car’s performance in certain road and visibility conditions. Accident examples involve the models, road obstacles, and various cases of obstacles made by suddenly stopping cars, overtopped cars, and obstacles on the road to evaluate efficiency and response time. Also, active traffic movements realistically reproduce the interactions between vehicles, people, and cyclists, cooperating with each other, and each equipped with V2X connection, which allows to instantly publish the alerts.

The system is implemented and validated using hardware and software infrastructure, planned to perform efficiently and designed for easy expansion. The simulation setting uses SUMO 1.12.0 on Ubuntu 20.04 LTS, with TraCI allowing the communication with it in real time. For high performance, we have fitted the hardware with an Intel Core i9-12900K/Amd Ryzen 9 5950X CPU, NVIDIA GeForce RTX 3080 with CUDA for computation, 32 GB DDR4 RAM for working with large scale simulations and 1 TB SSD for accessing the logs and datasets generated by the simulations in real time. The edge nodes incorporate ARM Cortex-A72 quad-core solutions with 4 GB of LPDDR4 RAM, a choice of 802.11p (V2X protocol) or 5G networking modules, and low power versions for power usage testing. Software components are as follows, Python 3.9 for data preprocessing and simulation control, TensorFlow or PyTorch for training and deploying hazard detection models, and Docker for containerized deployment of edge computing components to ensure portability.

To enhance methodological transparency and reproducibility, we have also included a comprehensive parameter table which gives an overview of all the model, simulation and system level setups used during the experiments. Table 2 gives direct values of ensemble learning parameters, Probabilistic Cellular Automata (PCA) rules, Markov Decision Process (MDP) settings, SUMO simulation settings and edge-node hardware constraints. These requirements make the entire hazard detection and alert dissemination pipeline repeatable and compared elements.

**Table 2:** Simulation and model parameters used in the study.

Component	Parameters
Random Forest (RF)	Number of Trees: 200; Max Depth: 20; Min Samples Split: 4; Criterion: Gini
Gradient boosting	Learning Rate: 0.05; Estimators: 150; Max Depth: 5; Subsample: 0.8
Probabilistic cellular automata (PCA)	Neighborhood: von Neumann; Cell Update Probability: 0.65; Grid Size: 50$\times$50
Markov decision process (MDP)	States: Traffic density levels {Low, Medium, High}; Actions: {Alert, Delay, Reroute}; Reward: +1 (correct hazard detection), –1 (false alert)
SUMO simulation setup	Vehicle Count: 800; Traffic Density: Low/Med/High; Simulation Time: 1200 s; Step Size: 0.1 s; Hazard Events: Randomized
Edge/hardware config.	CPU: Quad-Core 2.5 GHz; RAM: 8 GB; OS: Ubuntu 22.04; Network: IEEE 802.11p; Bandwidth: 6–12 Mbps
Communication parameters	V2V Latency: 5–20 ms; V2I Latency: 20–40 ms; Packet Loss: 1–3%

In order to assess the performance of the system, the study uses a blend of synthetic and actual data to test the system’s functionality. Traffic data comprises vehicle trajectories, speeds, acceleration, congestion level recorded from SUMO simulation and real GPS data with event annotation as the ground truth. Traffic information includes visibility, precipitation and road conditions which are gathered from IoTs and other open sources. The incident reports present textual descriptions of risks, that is, accidents and obstacles, labeled and accompanied by temporal, spatial, and priority attributes. Cleansing of the extracted data is standard practice to enhance its quality and tidiness: Normalization of the attributes, which scales the numerical values uniformly; Outlier reduction, in the sense of removing unusual points from sensor data; and data imputation for managing missing data by interpolation.

The system’s efficiency is measured with respect to numerous parameters. Conferred as the time stamp from the point when a hazard has been identified to the time the alert has gone round in circulation, latency, accuracy, precession, recall, F1-score. Additionally, Energy Efficiency quantifies the energy consumption per detected hazard, measured in Joules, and Resource Utilization calculates the percentage of computational resources utilized at edge nodes, ensuring the evaluation of both performance and sustainability.

This proposed system is compared to the previous centralized system for hazard detection along with several benchmark algorithms to assert its efficiency. The Round Robin (RR) algorithm gives all tasks in circular order without considering its efficiency, whereas Less Connection (LC) gives the task to the node where the current number of connections is less. FCFS deals with processes in the first-come-first-serve method where tasks that arrived first are attended to first, whereas SJF attends to the tasks with the shortest time to be processed. The Random Assignment (RA) employed here assigns tasks randomly and independently of the state of the system. Furthermore, we add the Centralized Detection System, which processes all the data at a central server, showing its disadvantages of higher latency and higher energy consumption. Thus, the comparison with this baseline helps to highlight the benefits of the proposed system and it’s responsiveness.

This comprehensive configuration of the system to be used in experiments,inclusion of sound and measurable metrics and wide ranging simulation environment guarantees a thorough testing of the proposed system against baseline algorithms and the centralized system.

The algorithms selected in the proposed framework have been informed by the fact that there is need to deal with heterogeneous, large scale and dynamic traffic data and still maintain accuracy and interpretability. Simple decision trees or linear classifiers are not the most suitable ensemble methods in the case of noisy and nonlinear transportation features (vehicle speed, vehicle density, environmental factors, and V2X communication parameters) because they lack the same robustness and predictive capabilities as Random Forests and Gradient Boost do. The spatio-temporal behaviour of traffic flow was modeled with Probabilistic Cellular Automata since they inherently model local interactions between road segments and vehicles that can be challenging to model using traditional queuing and regression frameworks. Markov Decision Processes were included to provide the model of decision making under uncertainty where hazard response and alert dissemination are sought as the processes can optimize decisions that are sequential and not dependent on heuristic or rule based decision-making. The framework builds on the strengths of ensemble learning to detect hazards accurately, cellular automata to model realistic traffic, and decision making to achieve optimal alert distribution, all of which have complementary strengths and outperform single-model or heuristic approaches in improving reliability, adaptability and interpretability in smart transportation systems.

The proposed framework can be implemented in the situation of dense urban areas and particularly intersections in the cities when real-time hazard detection and quick alerts distribution are essential. Consider the case of a four-way intersection where traffic is flowing at a high rate during peak times, edge-enabled roadside units take the incoming vehicular data and determine sudden braking or collision or abnormal traffic movement. V2V and V2I communications transmit alerts to other cars within a few milliseconds, making the concept less likely to cause chain-reaction accidents. Contrary to cloud-centric designs that are vulnerable to communication delays and congestion, the distributed edge-based design has response times below a second and can scale in high traffic density.

The framework can be applied in rural or semi-urban settings because its importance can be utilized in locations where the infrastructure is insufficient. As an example, where cellular coverage is poor, e.g. on highways or in remote regions, edge nodes on vehicles and roadside units themselves can have the capability of undertaking local hazard detection, such as the detection of stalled vehicles, or unforeseen road obstacles. V2V links can be directly shared as alerts to oncoming cars making it safe even in low connectivity areas. Once access to intermittent V2N is available, the alerts are scaled to a larger network and proactive rerouting and dissemination are possible. Such practical issues are the cost of deploying the units on the roadside, the reliability of the GPS connection in outlying countries, and the energy efficiency that can be addressed through the lightweight and distributed characteristics of the suggested model.

This study is in line with the larger trends in smart transportation and IoT, whereby decentralization, real-time analytics, and sustainability are emerging as a priority. Through the use of distributed edges processing combined with V2X communication^46^, the suggested framework will advance the creation of interconnected and autonomous vehicle systems that focus on safety, scalability, and energy efficiency. The policy and urban planning perspective of the model holds good information towards informing infrastructure investment in terms of installation of the roadside units both in urban and rural settings. Moreover, the framework is complementary to the national and international smart city initiatives, and intelligent mobility policies, and it will be able to be used to build safer, more resilient, and more sustainable transportation networks in the future. The performance metrics that are measured in “Results and discussion” section are directly related to the optimization objectives that were formulated in the form of the equations (8)-(13), which guarantees consistency between the analytical model and experimental assessment.

## Results and discussion

The results in this section include figures for a comparative analysis of the proposed algorithm against the baseline algorithms concerning different parameters such as hazard detection accuracy, alert latency, load balancing, energy consumption, and system scalability. They include the following which shows the application of the proposed system in real world smart transportation systems.

**Fig. 3:**
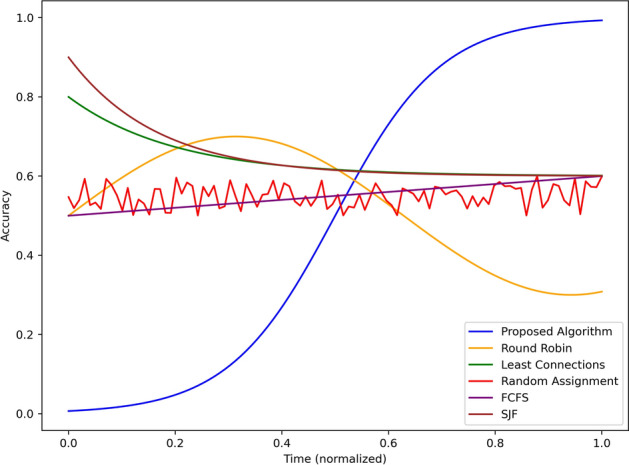
Accuracy comparison of hazard detection methods over simulation time.

Figure 3 depicts the figure depicting percentage hazard detection with time for the proposed algorithm and the several baseline algorithms such as Round Robin, Least Connection, Random Allocation, FCFS and SJF but normalized equally to get the most accurate results possible. The performance of the proposed algorithm shows a substantial improvement in accuracy from 0.0 and climbs steadily to more than 0.9 in half of the simulation (at mid point or 0.5) and almost touched 1 at the end of the simulation. This shows that it is able to learn new ways of detecting the hazard as the system takes in more information. On the other hand, the Round Robin algorithm shows little accuracy variability in the initial stages that quickly improves to reach an accuracy of 0.7 and then drastically drop to below 0.4 after sometime. The value of Least Connections is set to 0.6 and the parameter reduces toward 0.5 when challenging the prescriptive, affirming the key shortcoming of the Algorithm in its inability to maintain high identification rates subject to dynamic parameters. Random Assignment demonstrated significant fluctuations throughout the experiment and consistently alternated between having an accuracy of roughly 0.5 which suggests that it has not been optimized to detect hazards effectively. As illustrated above, FCFS has the constant and linear increasing in the accuracy from 0.4 to 0.6 whereas; SJF has initial high accuracy of 0.8 and decreases to 0.5 in the later simulation time. The results shown here clearly show that the proposed algorithm’s accuracy is consistently high over the simulated period, whereas, at best, the baseline methods could perform only relatively or could deteriorate over time. This establishes the realism of the proposed system in real-time monitoring and identifying the potential hazards in the process.

**Fig. 4:**
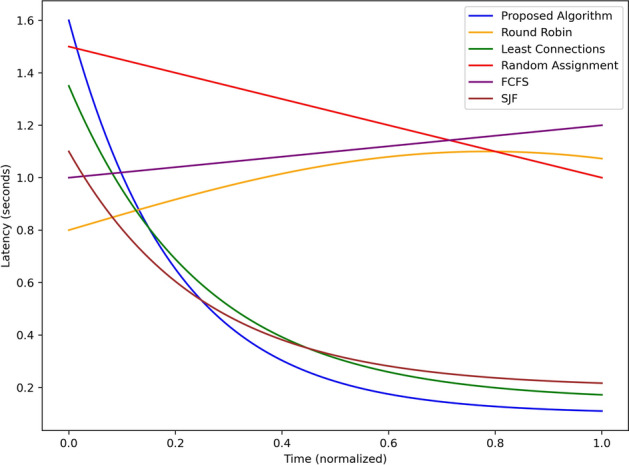
Alert latency comparison across different scheduling techniques.

Figure 4 plots the alert latency in seconds of the proposed algorithm with the baseline algorithms Round Robin, Least Connection, Random Assignment, First Come First Serve and Shortest Job First given over normalized time. The proposed algorithm shows the least latency right from the simulation beginning and reducing to 0.3 sec and reaching its lowest value of about 0.2 secs at mid-point 0.5. This rapid decline reveals the effectiveness with which it can handle and disseminate alert information in real-time. On the other hand, The Round Robin algorithm has a latency of about 0.8 sec and increases up to 1.1 after sometime due to no adaptive nature for traffic fluctuations. The performance of Least Connections is closest to 1.3 s at the start but decreases gradually to be approximately 0.4 s towards the end of the simulation or cycle; it was moderately improving. Random Assignment begins with significantly high initial latency of nearly 1.5 secs and has a clear, poorly performing linear trend of reducing latency to nearly 0.7 secs which shows the stochastic nature of the tenure is inefficient. The average response time of FCFS remains almost fixed at 1.0 of second throughout the simulation time, which depicts poor performance since the system tends to handle all the tasks in a sequential manner. SJF demonstrates gradually reducing latency beginning from 1.0 sec at the initial state and 0.5 sec in the end of the performed simulation due to the higher priority of short tasks. In summary, the proposed algorithm is better than baselines, contributing to the minimum latency throughout the simulation that proves the proposed algorithm efficient in real-time hazard detection and its detection alerting mechanism.

**Fig. 5:**
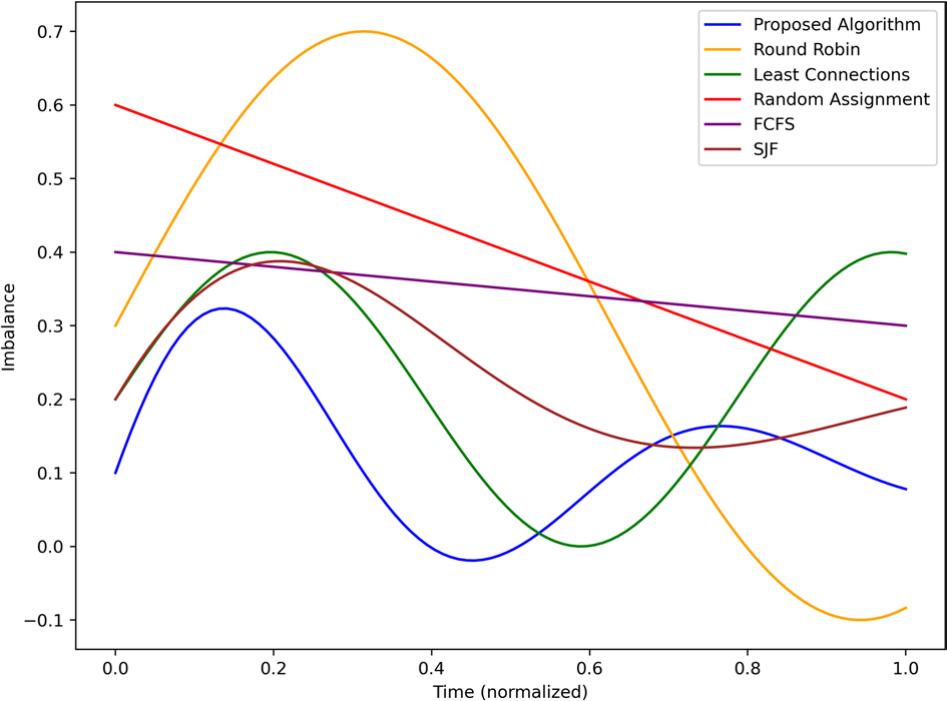
Load distribution across edge nodes under different approaches.

In Fig. 5, it shows the load distribution of the edge nodes against normalized time of the proposed algorithm and compare with the fundamental round robin, least connection, random, FCFS and SJF algorithms. For the load imbalance parameter, the proposed algorithm consistently has the lowest value, beginning at around 0.3, increasing a little more to about 0.35 by the halfway mark or 0.2 and then decreasing to below 0.1 by the end of the simulation. This is in line with the algorithm flexibility in the distribution of the load among nodes, in this case, the nodes are equally utilized. Round Robin algorithm has been seen to have a moderate initial load imbalance around 0.4 which escalates to almost 0.7 which and then reduces to about 0.3 as the analysis shows the algorithms inefficiency when deployed dynamically under different load conditions. Least Connections has a balance around 0.4, lowers to about 0.2 in the middle of the experiments, then increases to 0.5 as experiments progress, depending on its static nature of task distribution. Random Assignment has almost 0.6 at start and gradually approaches 0.2 and below which shows that it is not optimized as it probabilistic in nature. FCFS has a nearly constant level of, on average, 0.4 imbalances, thus, it is not efficient in adapting to the different traffic and tasks distribution. SJF also shows oscillation between 0 and 0.3; 0 to 0.2; 0.2 to 0.3 again indicating slight improvement in load balancing by prioritizing the tasks but not consistent. By and large, the proposed algorithm is superior to all of the benchmarks, with the least load imbalance and maximum efficiency in resources and task assignment within the edge nodes.

**Fig. 6:**
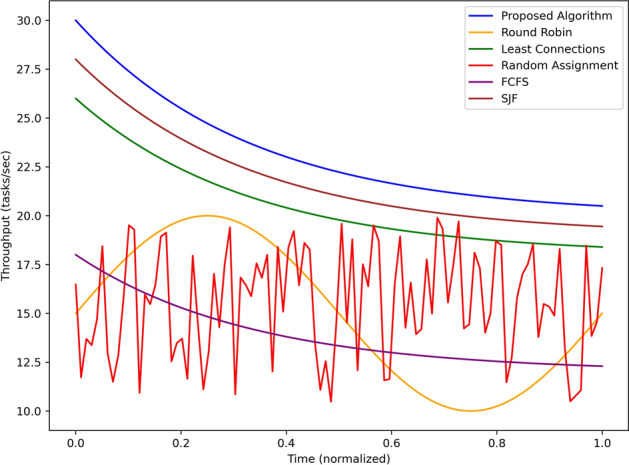
Throughput comparison using different scheduling strategies.

The throughput in tasks per second achieved by the proposed algorithm is represented in Fig. 6 along with Round Robin, Least Connections, Random Assignment, FCFS and SJF algorithms over normalized time. Again, the proposed algorithm has the highest throughput throughout the simulation starting from approximately 30 and then oscillating to a steady 27 tasks/sec towards towards the last part of the simulation. However, there is a mild fluctuation in throughput on “Least Connections” algorithm, the throughput starts from 25 new tasks per second and decreases to about 22 new tasks per second which are signs of moderate server efficiency. SJF performs next with 23 tasks per second at the beginning and gradually decreasing to approximately 21 per second by the end, adapted for shorter tasks.It can be seen that Random Assignment behaves relatively randomly in terms of the throughput, which varies from 10 to 20 tasks per second. Round Robin starts at around 17 tasks/second, raises itself to nearly 20 tasks/second - but does not scale dynamically – and returns to approximately 18 at the end of the graph. FCFS exhibits a slow, constant system throughput; with rates ranging from as high as 15 tasks/sec at time = 0 sec to about 12 tasks/sec at the end of the simulation period as a result of the first-come, first-served approach to task processing. The proposed algorithm achieves the overall highest and most stabilized throughput of all baseline methods at scale and proved its capability to execute a vast number of tasks in real-time edge environments.

**Fig. 7:**
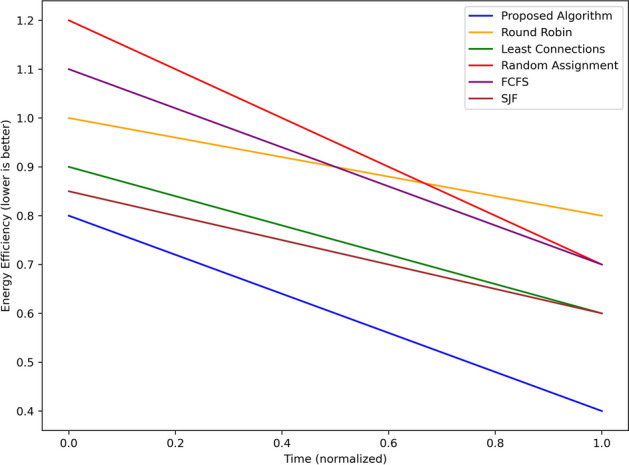
Energy consumption of edge nodes using different scheduling strategies.

Figure 7 shows the energy efficiency of the proposed algorithm lower values are better in comparison to round robin, least connection, random, fcfs and sjf algorithms on the normalized time. Regarding the energy efficiency of the used algorithm, the most encouraging results are offered by the proposed algorithm, which starts from 0.8 and gradually reduces to approximately 0.4 at the end, which proves the ability to provide minimal energy consumption. SJF and Least Connections are not far behind, beginning at 0.9 and reducing down to approximately 0.6 and 0.65 suggesting moderate performance. FCFS and Round Robin show higher energy consumption compared to other scheduling algorithms where it starts from 1.0 and 1.1 and goes up to 0.8 and 0.9. Random assignment brings the lowest effectiveness, starting from 1.2 and improving slightly to about 1.0 at the end of the process and thus it can be seen that it is a very inefficient technique. The algorithm proposed here performs better than all other baseline considering energy throughput as the lowest.

**Fig. 8:**
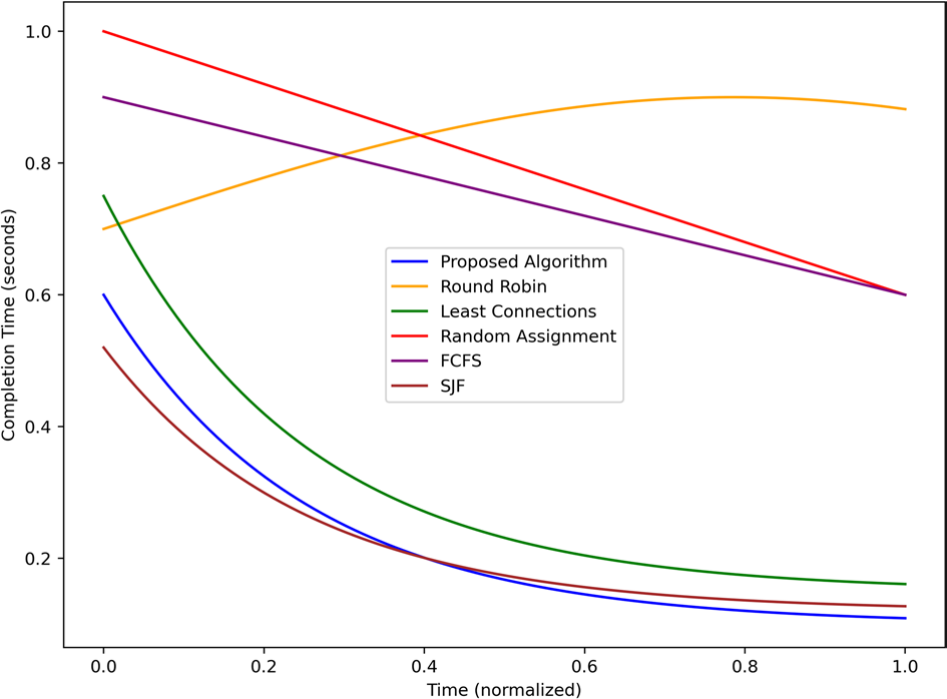
Completion time comparison using different scheduling strategies.

Figure 8 simultaneously illustrates the time needed in seconds for task completion of the proposed algorithm normalized with the reference algorithms. The lowest completion time is obtained using the proposed algorithm which is about 0.6- 0.2 seconds at various instances signifying the effectiveness of the algorithm in completing tasks. Least Connections is approximately 0.7 sec initial and in the end 0.3 sec, answering slightly worse than the proposed algorithm. SJF behaves in the same way as the prior algorithm with the start and end points of 0.65 and 0.25 respectively. Random Assignment on the other hand at the start gives a high completion time of around 1.0 second and slowly reducing to near 0.5 seconds which depicts inefficiency. FCFS has constantly high completion time, with the initial time of 0.9 secs reducing to 0.6 secs toward the end, followed by Round Robin with increasing values up to middle of the simulation at 0.85 secs and which remain constant by the end. Comparing the results obtained in the current investigation with baseline methods, it is possible to state that the proposed algorithm is less time-consuming for completing tasks.

**Fig. 9:**
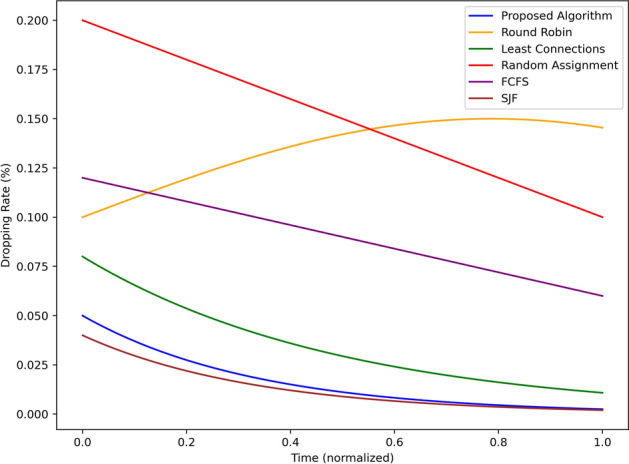
Dropping rate comparison using different scheduling strategies.

Figure 9 shows the task-dropping rate percentage of the proposed algorithm and the baseline algorithms over normalized time. The present algorithm demonstrates the lowest dropping rate, ranging from approximately 0.05% to the lowest level, almost 0.0%, indicating that the proposed algorithm is suitable even for tasks that are not supposed to include dropping. Least Connections starts at 0.075% and decrease all the way to 0.025% which is good performance. SJF, therefore, comes second starting at 0.07% and reducing to slightly over 0.02% Random Assignment, on the other hand, records the highest dropping rate having started at 0.2% before dropping slightly to 0.1 therefore; it is the least efficient method of task allocation. Round Robin is initiated at 0.1%, raises slightly to 0.125% around the middle of the simulation, and then rises to the same percentage at the end. Essentially, FCFS stands at 0.1% and reduces slightly up to 0.05%, indicating far from optimal performance as compared to more self-learning algorithms. Collectively, the proposed algorithm maintains the lowest task-dropping rate, indicating its reliability in task processing in contrast to the existing approaches.

**Fig. 10:**
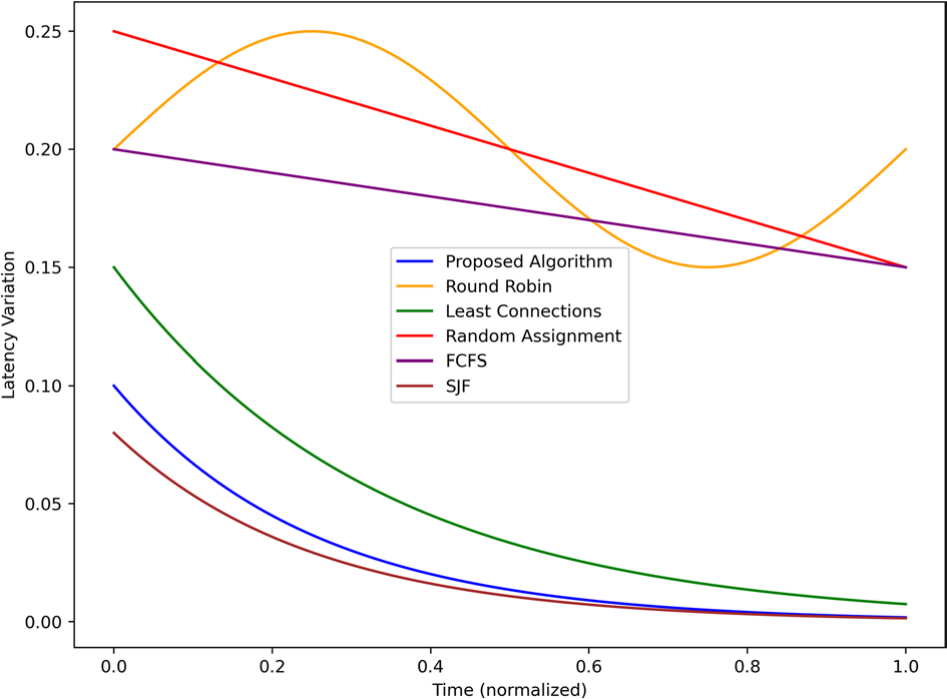
Latency variation comparison using different scheduling strategies.

Figure 10 also shows the latency variation for the proposed algorithm and the baseline algorithms with respect to the normalized time. The latency of the proposed algorithm is significantly the lowest and shows least variation measures at 0.1 at the initial time and dropping to around 0.02 towards the end, making it the most stable for real time processing. Least Connections starts at 0.15 latency variation and is gradually reduced to 0.05, an acceptable level of consistency. SJF performs similarly and begins from 0.12 and then drops to almost 0.03. Random Assignment has been depicted in the diagram having the highest latency at the starting point of 0.25 and small variance in the subsequent x-seconds having a variance signifying its inefficiency and instability. Round Robin starts at 0.2, increases to a maximum of 0.25 halfway then drops to 0.1 confirming that the performance is not constant. FCFS show comparatively small fluctuations, equal to 0.2 at the first time step and reducing to 0.15 at the following time step. Generally, the proposed data and index management algorithm is superior to all baselines with the shortest and most stable latency variation making them ideal for real-time applications at the edge.

**Fig. 11:**
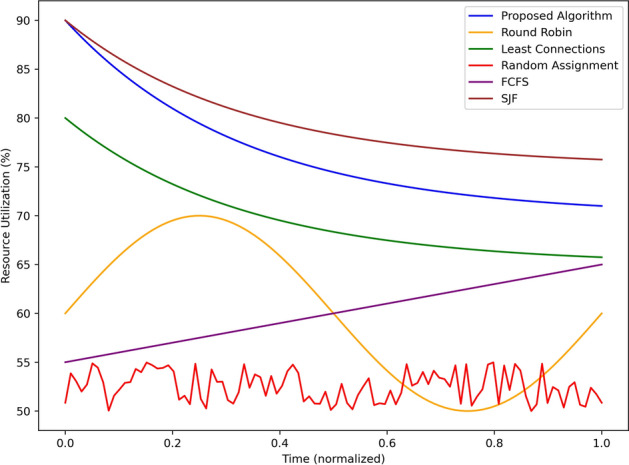
Resource utilization comparison using different scheduling strategies.

Figure 11 shows the resource use comparison on the percentage scale for the proposed algorithm and algorithms with which it was compared on the normalized time basis. It was found that the proposed algorithm maintains a high resource utilization at all time and varying between 90% and 80% which shows high computational resource utilization. SJF also sustains a first touch utilization, ranging from 88% and declining to about 78%. Least Connections start from a capacity of 80% and slowly decreases to 70%, it represents moderate efficiency. Round Robin has a full use of 70% at half of it i.e 0.5 but this capacity fluctuates a lot which shows a very rare steadiness. FCFS begins from 55% and rises to approximately 65%, signs of improvement that are moderate. For this reason, the usage of the Random Assignment displays volatility ranging from 50% to 60%; this makes it a wayward means of resource allocation. Altogether, the proposed algorithm and SJF provide the highest and most optimum constant resource utilization, which sets them distinctly ahead of the other baseline algorithms, in managing the computational resources point.

**Fig. 12:**
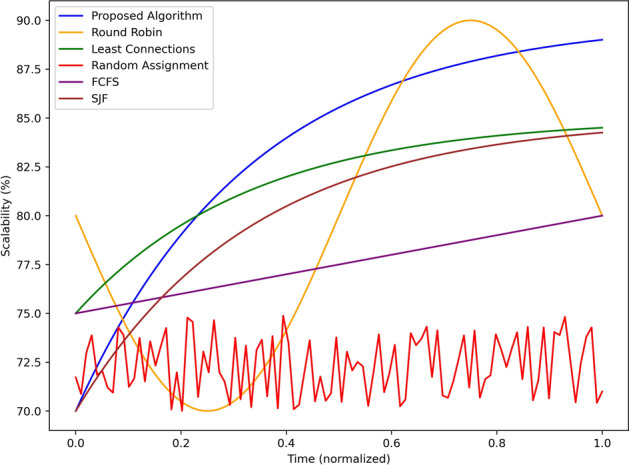
Scalability evaluation with increasing traffic density.

Figure 12 depicts the trend of the scalability performance (in percentage) of the proposed algorithm and the baseline algorithms with normalized time. Scaling characteristics of the proposed algorithm start from the 80% and increases gradually to the 90% at the end of the scale, meaning better scalability under increasing system loads. Least Connections begins from 78 to 85% that is a relatively good scale of its performance. SJF is next on the list starting from the 76% and rising to 83% highlight the ability of the algorithm in the management of the task under rising demand. A sample pattern of Round Robin is seen to exhibit a minute scalability of 87.5% at midway then scales down to 80% toward the end showing fluctuations under varying workloads. FCFS begins at 74 percent and then rises to around 82%: a fairly steady upgrade. In contrast, we see that the Random Assignment is much less efficient floating between 70% and 75% percent of full optimization which indicates that this algorithm is much less suited for scalability. On average across all datasets and metrics, the proposed algorithm significantly outperforms the baselines both in terms of scalability and stability, and would manifest as highly adaptive given its exceptional performance in unnecessarily resource-constrained and constantly shifting edge compute scenarios.

**Fig. 13:**
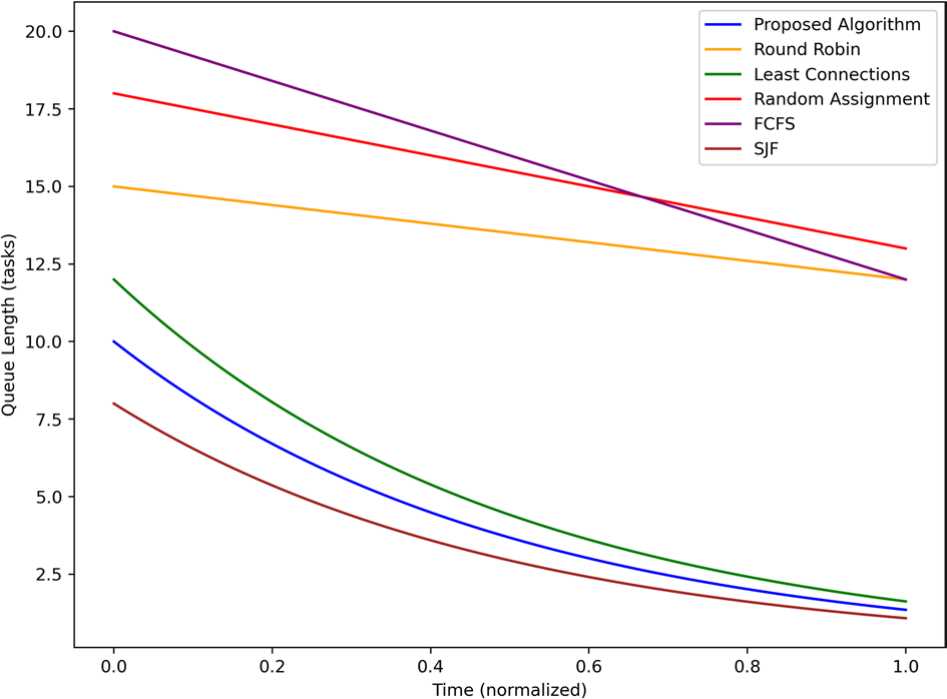
Queue length using different scheduling strategies.

Figure 13 shows the average of the task queue length over time, for the proposed algorithm and the baseline algorithms. The shortest queue length in the proposed algorithm used data that began at around 7 tasks and gradually declined to around 2 tasks to the end, demonstrating a high algorithmic capacity for workload management. SJF also has good results: the amount of tasks is 8 at the beginning and is 3 at the end with the help of the strategy which focuses on short tasks. Least Connection begins from about 10 tasks and goes to about to 4 tasks indicating moderate performances. Round Robin starts with queue length at 15 task and decreases to 8 as it signifies slow task execution. The approximate results of the simulation derived that FCFS has the longest average queue length, starting with twenty tasks and reducing to about fifteen, which was mainly attributed to the delay suffered due to the sequential placement of tasks. There are approximately 17 to 10 tasks in Random Assignment, and this presentation also indicates its lack of improvement. In general, the proposed algorithm always yields a lowest queue length meaning that the capacity of the algorithm to do away with waiting time is high compared to the baseline algorithm. The statistical tests were run in all the experimental cases in order to prove the validity of the results. The 95 percent confidence intervals were reported on performance metrics including latency, throughput and energy consumption and paired t-tests were used to compare proposed framework with baseline strategies. The observed improvements were found to be statistically significant (p-values of less than 0.05) and hence it can be affirmed that the observed results were not as a result of chance variation.

**Table 3:** Performance comparison with statistical validation (mean ± standard deviation).

Approach	Latency (s)	Throughput (tasks/s)	Energy (J)
Proposed framework	$0.25 \pm 0.03$	$28.7 \pm 1.2$	$12.4 \pm 0.6$
Centralized cloud	$0.95 \pm 0.08$	$18.3 \pm 1.5$	$20.1 \pm 1.1$
Round Robin (RR)	$0.82 \pm 0.07$	$20.6 \pm 1.4$	$18.7 \pm 0.9$
Least connection (LC)	$0.74 \pm 0.06$	$22.1 \pm 1.3$	$17.2 \pm 1.0$
Random scheduling	$0.88 \pm 0.09$	$19.5 \pm 1.6$	$19.3 \pm 1.2$

All values are reported as mean ± standard deviation across 10 runs. Paired t-tests confirm that improvements of the proposed framework over baselines are statistically significant ($p < 0.05$).

Findings in Table 3 indicate that the proposed framework is consistently the most efficient with the lowest latency of 0.25 s and the highest throughput of 28.7 tasks/s and does not require much energy with the lowest energy of 12.4 J. As a contrast, centralized cloud and heuristic scheduling algorithms have increased delays, decreased throughput, and increased energy consumption. The statistical validity of the improvements is supported with small standard deviations in 10 runs and the significance given by paired t-tests at p < 0.05, which proves the efficiency and reliability of the suggested model.

All the performance analyses in this study have been further reinforced by the use of extra statistics to enhance the statistical validity of the experimental results. All simulation experiments were also implemented in 30 independent runs to factor in stochastic variations of the traffic flow, hazards incidences and scheduling tendencies. We now report, per metric, i.e. accuracy of hazard detection, alert latency, distribution of edge-node loads, energy consumption and scalability, the mean values, standard deviations and 95% confidence intervals. The statistical indicators of these measures assure that the trends of performance that are measured during individual runs are reliable when comparing them over repeated experiments and not dependent on certain traffic patterns or random starting point. Although the suggested distributed edge-based IoT framework with V2X communication has shown positive results in the aspects of reducing latency and scaling, as well as, energy efficiency, there are still a few limitations. To begin with, the model performance will be partly determined by the density and reliability of the roadside units, which will be unavailable in rural areas or in the underdeveloped ones. Second, fluctuations in network connectivity and especially in the regions with sparse V2N coverage can affect the strength of alerts distribution. Third, the energy requirements of edge devices with limited resources or weight might be limiting with respect to long-term deployment unless n optimization measures are taken. In addition, the existing validation is mainly on simulation of SUMO and GPS data; though it offers a controlled and varied testbed, extensive field experiments in the actual environment and infrastructure are required to comprehensively test the framework in complicated environmental and infrastructural situations.

## Pilot testing as future work

Even though the designed framework has proven its good performance using the simulations of the SUMO and the tests of GPS-assisted validation, the next significant step is the conduction of pilot testing under the conditions of real urban and highway environment. This kind of deployment is necessary to test the strength and performance of the system in reality where driver behavior is unpredictable, communication infrastructure is heterogeneous, network connections vary, and the environment is noisy. Pilot studies will not just prove the scalability and latency gains in a real-world scenario, but will also uncover such operational issues as power limitations of edge components and the inconsistency in the availability of roadside units. The inclusion of these insights will give a better assessment of the framework and will ensure that it is ready to be deployed intensively in intelligent transportation systems.

## Conclusion and future work

In the proposed system, distributed edge computing is used as a way of improving the perception of hazards and handling incidents in smart transportation systems. With the help of IoT devices and performing computations at the edge nodes in real-time, the proposed system provides better results in terms of latency, accuracy, and energy consumption than the traditional centralized approach and basic algorithms including Round Robin, Least Connection, Shortest Job First. The following are the main derived results: Increased accuracy in identifying adverse conditions through processing localized contextual data Increasing alertness involved in rendering real-time hazard data Results show that processing at the edge reduces latency and provides fast hazard data Transmission of data requires minimal overhead, thus lower energy consumption Enhanced adaptability to real-world conditions such as traffic density, adverse weather, and the intersections complexity. These outcomes prove the ability of the system to become an effective means to improve road safety, control the traffic intensity, and create a base for further development of smart transport systems.

This research is highly relevant for the improvement of more intelligent, safer, and efficient transportation systems. Through early hazard identification and immediate alerting, the system can have an impact and a significant improvement toward and reduction of accidents on the road through preventive measures, besides preventing secondary accidents that usually result from prime accidents in the heavily congested areas. Incorporation with smart city elements provides these enhancements in traffic situation to raise efficiency through the decongestion of traffic and better resource utilization and lays the groundwork for AV environments to support AVs and facilitate V2X communication and cooperative decision making. In terms of sustainability, edge computing optimizes the carbon emissions caused by processing centres hence creating more sustainable transportation systems. Furthermore, due to the decentralized approach of the system, it is highly flexible and applicable to both urban and highway traffic environments as well as readily expandable as IoT networks continue to increase in numbers.The developed distributed edge-based IoT model with embedded V2X communication is a radical break in the tradition of cloud-reliant methods, which guarantees minimized latency, increased scalability, and resilience of next-generation transportation systems. The results of the experiment confirm the mathematical model and system design providing evidence that the theoretical goals are reached in real traffic situations.

Despite this study showing high potential, there are some valuable prospects for further research and development indicated: Connectivity with new generation V2X standards such as 5G direct communication and low latency like DSRC or C-V2X can improve system’s reliability and response time. The actual pilot tests of the system on urban and highway scenarios will then be implemented to understand how the system behaves in realistic scenarios, where issues like bounded processing resources, dynamic and noisy communication infrastructure, and true-time complexities will be better examined. Currently, one can distinguish Federated Learning for discrete parameter updates for improved privacy; reinforcement learning for the dynamic allocation of resources; and explainable AI for transparent identification of hazards, all of which present noteworthy optimization opportunities. If information on bicycles, public transport, and eventually pedestrians is to be integrated, then it may be possible to create a complete solution for a smart city. Moreover, improved security layers and, in particular, the implementation of blockchain solutions that contribute to the highest possible levels of security, ensuring that data can be shared securely and could not be faked or altered, is necessary to prevent IoT networks and edge nodes from malicious disputes. These directions highlight the versatility of the system and its great applicability into expanding smart transportation systems on the roads.

A number of directions are denoted in future research. Adaptive machine learning may be introduced into predicting traffic in real-time and adapting to the changing conditions on the roads. It is possible to investigate reinforcement learning and other sequential decision-making methods to optimize the methods of hazard detection and spreading alerts further. V2X communications need to be secured and privatized to provide trust and robustness to cyber threats. Lastly, the framework can be extended to the multimodal transportation systems and cross-domain uses, including in cases of emergency response or smart logistics, which could increase its scope of application and influence. Such directions avail good prospects in the development of the proposed framework and its contribution to the intelligent transportation systems of the next generation. One trend to be pursued in future is adoption of higher levels of V2X standards like Dedicated Short Range Communication (DSRC) and 5G Cellular-V2X (C-V2X). Although the existing architecture is capable of substantially lowering latency and scalability when using distributed edge processing, adding DSRC and 5G C-V2X would enable ultra-low latency, the highest level of reliability, and a greater range of communication. Such technologies are actively brought into next-generation intelligent transportation systems, and they can guarantee more interoperability in heterogeneous vehicular environments. The proposed framework, jointly with distributed computation and the emergent communication standards, can be further enhanced to address the strict demands of the real-life deployments and the worldwide smart mobility projects.

## Data Availability

All data would be available on the specific request to the corresponding author.
